# The survey of electrophysiology lab activity during the COVID‐19 pandemic

**DOI:** 10.1002/joa3.12584

**Published:** 2021-06-24

**Authors:** Javad Shahabi, Mozhde Askari, Amirhossein Azhari, Mohammad Kermani‐Alghoraishi

**Affiliations:** ^1^ Interventional Cardiology Research Center Cardiovascular Research Institute Isfahan University of Medical Sciences Isfahan Iran; ^2^ Hypertension Research Center Cardiovascular Research Institute Isfahan University of Medical Sciences Isfahan Iran

**Keywords:** COVID‐19, EP lab, implantable cardioverter defibrillator, permanent pacemaker

## Abstract

**Background:**

With the onset and spread of the COVID‐19 pandemic, the hospitalization and treatment of noncovid patients were dramatically affected. The aim of this study is to evaluate the electrophysiology (EP) lab activity in a referral center in Iran during the COVID‐19 era.

**Methods:**

A cross‐sectional descriptive survey was conducted on EP lab activity in Shahid Chamran Heart Center, Isfahan, Iran. Two periods of COVID‐19 occurrence peaks in Iran were compared with same date in 2019. Information was collected on number of diagnostic and therapeutic electrophysiology studies (EPSs) and implantation of intracardiac devices such as permanent pacemaker (PPM), implantable cardioverter defibrillator (ICD), and cardiac resynchronization therapy (CRT).

**Results:**

In the first peak of COVID‐19 pandemic, both of EPSs and intracardiac device implantations decreased by 80% compared to the same period in 2019. The most common type of device implanted during this period was PPM (70%); however, at the time of control, the ICD (73%) was the most common. Paroxysmal supraventricular tachyarrhythmia (PSVT) was the best indication for diagnostic and therapeutic EPSs in covid and control periods. In the second peak of prevalence of COVID‐19 virus infection in Iran, 6% and 36% decreases in device implantations and EPSs were seen, respectively. During this period, the number of procedures increased, although it was still lower than in 2019.

**Conclusion:**

A significant reduction in the EP lab activity has been observed during both the COVID‐19 pandemic peaks.

## Abstract 2

This study is a cross‐sectional descriptive survey conducted on EP lab activity in Shahid Chamran Heart Center, Isfahan, Iran (as largest Heart Center in center of Iran), regarding number of EPS and intracardiac device implantations during the two peaks of COVID‐19 pandemics in Iran. The same date in 2019 was considered as control time. In conclusion, our EP lab activity decreased significantly during the COVID‐19 era.

## 1 INTRODUCTION

A severe acute respiratory syndrome coronavirus 2 (SARS‐CoV‐2) named COVID‐19 broke out in December 2019, in Wuhan, China, and spread rapidly around the world.^1^ Many health and therapeutic protocols have been changed with the announcement of a pandemic by the World Health Organization (WHO) and the growing prevalence of the disease worldwide. Initially, the main purpose of these protocols was to reduce the exposure of medical staff to SARS‐Cov‐2 virus as well as reduction in nonemergency diagnostic and therapeutic procedures to take maximum advantage of structural and functional capacity of hospitals.^2^, ^3^, ^4^ However, with the prolongation of the pandemic and increasing recognition of the nonpulmonary complications of COVID‐19, such as cardiovascular disease, the need to return all health services of hospitals was felt. Among these, cardiac electrophysiological studies (EPSs) and implantation of intracardiac devices were significantly decreased during the COVID‐19 era; however, increased incidence of cardiac arrhythmias in COVID‐19‐infected patients, greater incidence of ischemic (because of delay for appropriate treatment) and nonischemic cardiomyopathies, and economic and social stress arising from quarantine rules are reasons why these procedures are necessary.^5^, ^6^, ^7^, ^8^

Statistics and information about the activity of electrophysiology laboratory (EP lab) did not publish much during the COVID‐19 era, so given the preventive importance of this issue, we seek to provide data about our EP lab activities regarding diagnostic and therapeutic procedures in COVID‐19 pandemic period and compare it with non‐COVID‐19 period.

## 2 METHODS

### 2.1 Study design

A cross‐sectional descriptive survey on EP lab activity in Shahid Chemran Heart Center, as a largest referral hospital in central of Iran, was conducted. The average number of diagnostic and therapeutic EPSs and implantation of intracardiac devices such as permanent pacemaker (PPM), implantable cardioverter defibrillator (ICD), and cardiac resynchronization therapy (CRT) is 1000 cases per year in mentioned center. This survey was referred back to two durations of COVID‐19 pandemic peak in Iran: first peak between 5 March 2020 and 20 May 2020, and second peak between 21 June 2020 and 21 September 2020. The same date in 1 year ago (2019) was defined as control period. All data were collected during the 10 days from 25 September to 5 October from hospital registry, electrophysiology department. Because of all data were anonymized, ethical approval was not required.

### 2.2 Variables

Our main objectives were quantity of diagnostic and therapeutic EPSs and intracardiac device implantations procedures in the first and second COVID‐19 peaks in Iran in comparison to pre‐COVID‐19 condition. In the first peak, we experienced restricted quarantine rules for general population and health centers, whereas in the second courier, quarantine rules became more limited. Medical indications of these procedures are mentioned as secondary variables.

### 2.3 Statistical analysis

Quantitative data were analyzed as mean and standard deviation, whereas qualitative data were presented as number and percentages. To compare quantitative data between the two mentioned times, independent *t* test was used and, for qualitative data Chi‐square test and, if necessary, Fisher's exact test was used. *P*‐value below 0.05 was considered as a significant level. Statistical Package for the Social Sciences (SPSS) version 24.0 was used to analyze the data.

## 3 RESULTS

In the first peak of COVID‐19 pandemic, 10 patients were under intracardiac device implantation and 11 patients were under EPS, whereas in the same time of this period 48 patients were under intracardiac device implantation and 55 patients were under EPS (80% decrease). There was no significant difference between first COVID‐19 pandemic and control period based on age and gender in the patients under device implantation (*P* > .05). In the first peak of coronavirus outbreak, PPM implantation as a result of atrioventricular node or sinoatrial node diseases (40%) was the most common reason for implantation of intracardiac device (70%), while in the same period in 2019, ICD implantation because of cardiomyopathies (35.4%) was the most common (73%). In general, there was a significant difference in the number intracardiac device implantation between the COVID‐19 and noncovid era (*P* = .03) (Table 1).

**TABLE 1:** Variables of study in patients under intracardiac device implantation in the first peak of COVID‐19 pandemic

Variables		COVID‐19 pandemic period	Control period	Variation %	*P*‐value
Total number of intracardiac device implantation		10	48	−80	‐
Gender (male/female)		4/6	34/14	‐	0.06
Age (mean ±SD) (years)		61.22 ± 14.13	57.22 ± 17.50	‐	0.52
Indication for PPM implantation	AF with bradycardia	0	1 (2.1%)	−100	0.03
	SAN diseases	1 (10%)	1 (2.1%)	0	
	AVN diseases	6 (60%)	7 (14.6%)	−14	
Indication for ICD/CRT implantation	LV dysfunction	1 (10%)	9 (18.8%)	−89	
	ERI ICD/CRT	1 (10%)	9 (18.8%)	−89	
	ICMP, DCM, HCM	1 (10%)	17 (35.4%)	−94	
Other indications	High threshold RV lead/RV lead dislodgment	0	4 (8.3%)	−100	

Abbreviations

AF, atrial fibrillation; AVN, atrioventricular node, CHB, complete heart block, CRT, cardiac resynchronization therapy, DCM, dilated cardiomyopathy, ERI, elective replacement indicator, HCM, hypertrophic cardiomyopathy, ICD, implantable cardioverter defibrillators, ICMP, ischemic cardiomyopathy, LV, left ventricle, PPM, permanent pacemaker, RV, right ventricle, SAN, sinoatrial node.

For the patients under diagnostic and therapeutic EPSs, the most common cases in first COVID‐19 pandemic peak and control period were paroxysmal supraventricular tachyarrhythmia (PSVT) including atrioventricular nodal reentry tachycardia and atrioventricular reentry tachycardia (63.6% and 38.1%, respectively). There was no significant difference between first COVID‐19 pandemic peak and control period based on gender and EPS indications (*P* > .05). The mean age in the first COVID‐19 pandemic was significantly higher than control period in patients under EPSs (*P* = .02) (Table 2).

**TABLE 2:** Variables of study in patients under EPS in the first peak of COVID‐19 pandemic

Variables		COVID‐19 pandemic period	Control period	Variation %	*P*‐value
Total number of EPS		11	55	−80	
Gender (male/female)		5/6	23/32	‐	0.53
Age (mean ±SD) (years)		61.45 ± 15.46	49.72 ± 15.49	‐	0.02
Indication of EPS	PSVT (AVNRT, AVRT)	7 (63.6%)	21 (38.1%)	−67	0.59
	Atrial tachyarrhythmia (AT, AFL, AF)	1 (9.1%)	9 (16.3%)	−89	
	Bradyarrhythmia (SAN and AVN diseases)	1 (9.1%)	6 (10.9%)	−84	
	PVC/VT	0	6 (10.9%)	−100	
	Others	2 (18.2%)	13 (23.6%)	−85	

Abbreviations

AT, atrial tachycardia; AF, atrial fibrillation; AFL, atrial flutter; AVN, atrioventricular node; AVNRT, atrioventricular nodal re‐entry tachycardia; AVRT, atrioventricular re‐entry tachycardia; CHB, complete heart block; EPS, electrophysiology study; PSVT, paroxysmal supraventricular tachyarrhythmia; PVC, premature ventricular contraction; SAN, sinoatrial node; VT, ventricular tachycardia.

In the second COVID‐19 pandemic peak, 123 patients were under intracardiac device implantation and 77 patients were under EPS and, at the same time of this period in the past year (control period), 131 patients were under device implantation and 121 patients were under EPS. The number of cases in the second COVID‐19 pandemic period was decreased 6% for intracardiac device implantation and 36% for EPS compared to control period. There was no significant difference between second COVID‐19 pandemic peak and control period based on age and gender in patients (*P* >.05). The most common indication for device implantation in second peak of COVID‐19 pandemic and same control period was cardiomyopathy (41.9% vs 29.5%). During this period, there was also a significant difference between the coronavirus period and control time in terms of the total number of intracardiac devices implantation (*P* =.008) (Table 3). The most common indication for EPSs in both second COVID‐19 pandemic peak and control period was PSVT as well (45.4% and 53.7%, respectively) (Table 4).

**TABLE 3:** Variables of study in patients under intracardiac device implantation in the second peak of COVID‐19 pandemic

Variables		COVID‐19 pandemic period	Control period	Variation %	*P*‐value
Total number of intracardiac device implantation		123	131	−6	
Gender (male/female)		79/45	90/42	‐	0.26
Age (mean ±SD) (years)		62.92 ± 15.05	64.53 ± 14.45	‐	0.38
Indication of PPM implantation	AF with Bradycardia	1 (0.8%)	0	‐	0.008
	SAN diseases	2 (1.6%)	4 (3%)	−50	
	AVN diseases	26 (21.1%)	32 (24.4%)	−19	
Indication of ICD/CRT implantation	LV dysfunction	19 (15.3%)	20 (15.2%)	−5	
	ERI ICD/CRT	20 (16.1%)	35 (26.5%)	−43	
	ICMP, DCM, HCM	52 (42.2%)	39 (29.5%)	+33	
Other indications	High threshold RV lead/RV lead dislodgment	3 (2.4%)	1 (0.8%)	+200	

Abbreviations

AF, atrial fibrillation; AVN, atrioventricular node, CHB, complete heart block, CRT, cardiac resynchronization therapy, DCM, dilated cardiomyopathy, ERI, elective replacement indicator, HCM, hypertrophic cardiomyopathy, ICD, implantable cardioverter defibrillators, ICMP, ischemic cardiomyopathy, LV, left ventricle, PPM, permanent pacemaker, RV, right ventricle, SAN, sinoatrial node.

**TABLE 4:** Variables of study in patients under EPS in the second peak of COVID‐19 pandemic

Variables		COVID‐19 pandemic period	Control period	Variation %	*P*‐value
Number of EPS		77	121	−36	
Gender (m/f)		37/40	62/59	‐	0.38
Age (mean±SD)(years)		54.12 ± 19.66	52.15 ± 16.94	‐	0.45
Indication of EPS	PSVT (AVNRT, AVRT)	39 (50.6%)	70 (57.8%)	−44	0.08
	Atrial tachyarrhythmia (AT, AFL, AF)	10 (12.9%)	8 (6.6%)	+25	
	Bradyarrhythmia (SAN and AVN diseases)	12 (15.5%)	15 (12.4%)	−20	
	PVC/VT	1 (1.3%)	13 (10.7%)	−92	
	Others	15 (19.5%)	15 (12.4%)	0	

Abbreviations

AT, atrial tachycardia; AF, atrial fibrillation; AFL, atrial flutter; AVN, atrioventricular node; AVNRT, atrioventricular nodal reentry tachycardia; AVRT, atrioventricular reentry tachycardia; CHB, complete heart block; EPS, electrophysiology study; PSVT, paroxysmal supraventricular tachyarrhythmia; PVC, premature ventricular contraction; SAN, sinoatrial node, VT, ventricular tachycardia.

No COVID‐19 patients underwent diagnostic study in either peaks. Among the statistics provided, 1 patient (PPM) in the first peak and 11 patients (PPM: 10 cases, ICD: 1 case) during the second peak needed emergency intracardiac device implantation.

## 4 DISCUSSION

Based on our results, the number of cases for intracardiac device implantation and EPSs was decreased in the first COVID‐19 pandemic peak compared to the same time of the past year. This decrease was also evident in the second peak of pandemic; however, compared to the first peak, it was less different from the control period. The reduction in device implantation and EPSs in the first COVID‐19 pandemic was 80% for both, but in the second COVID‐19 pandemic peak were 6% for device implantation and 36% for EPSs. The point is that the decrease in EP lab activity in both COVID‐19 pandemic peaks is caused by this unprecedented epidemic in Iran, but the further decrease in device implantation and EPS cases in the first pandemic peak was as a result of the Ministry of Health's restrictions on services for elective patients and the lack of insurance coverage for these services. On the other hand, the social restrictions on dealing with the COVID‐19 pandemic were much greater in the first peak, so that most invasive and noninvasive procedures were canceled. In the second pandemic, most of the social and health restrictions were reduced. There is also the hypothesis that patients were afraid to go to medical centers during the pandemic, especially during the first peak. As the pandemic trend continued, although the EP lab activity increased, it was still lower than in the previous year, which could be because of the reluctance of physicians and medical staff to perform elective procedures, continued patients fear coming to medical centers, and financial incapacity caused by economic problems.

A similar study by Gonzales et al was done in Peru to investigate the effect of the COVID‐19 pandemic on the frequency of pacemaker implantation. The authors found that the COVID‐19 pandemic reduced 73% of de novo pacemaker implantation, and the number of diagnosing CHB in the COVID‐19 pandemic was reduced by 78%.^5^

In a similar survey, Li et al reviewed EP lab activity in three cities (Wenzhou in China, Milan in Italy, and London in the UK). They showed a significant reduction in EP lab procedures within a week of the recognition of widespread community transmission of the virus in each region. Their activity was dependent on new national COVID‐19 patient's diagnosis figures. In general, during the period of restrictions, their workflow had decreased to less than 5% of normal and included only emergencies.^6^

Ultimately, this survey showed that as more time elapses since the pandemic outbreak and as we acquire a sound understanding of principles of diseases prevention and treatment, the performance of medical centers gradually reverts to its standard form. However, the prevalence and severity of COVID‐19 in each region will determine the therapeutic approaches of the centers in each period.

## 5 LIMITATIONS

The single‐center assessment is the limitation of this study, although it is a large and referral hospital in central of Iran and can reflect the activities of other centers. Lack of information about the consequence of the patients for whom their diagnostic and therapeutic approaches, especially intracardiac devices implantation, were postponed or cancelled is the other limitation of this survey.

## 6 CONCLUSION

Based on the results of the present study and other surveys, COVID‐19 pandemic decreased the numbers of referred patients with cardiac problems for EPS or intracardiac device implantation to hospitals as patients/medical staff fearing to infect of COVID‐19 and/or low willingness of the health‐care system to provide these services.

## DISCLOSURES

All authors have no financial interests to disclose and no conflicts of interest to declare.

## CONFLICT OF INTEREST

None.
